# In vitro analysis reveals necroptotic signaling does not provoke DNA damage or HPRT mutations

**DOI:** 10.1038/s41419-020-02879-y

**Published:** 2020-08-13

**Authors:** Mark A. Miles, Christine J. Hawkins

**Affiliations:** Department of Biochemistry and Genetics, La Trobe Institute for Molecular Science, La Trobe University, Victoria, Australia

**Keywords:** Cancer prevention, Necroptosis

## Abstract

Most anticancer drugs provoke apoptotic signaling by damaging DNA or other means. Genotoxic therapies may enhance a patient’s risk of developing “therapy-related cancers” due to the accumulation of oncogenic mutations that may occur in noncancerous cells. Mutations can also form upon apoptotic signaling due to sublethal caspase activity, implying that apoptosis activating drugs may also be oncogenic. Necroptosis is a different way of killing cancer cells: this version of caspase-independent cell death is characterized by receptor-interacting protein kinase-3 (RIPK3) and mixed lineage kinase-like domain protein (MLKL) activation, leading to cell membrane rupture and controlled cell lysis. The mutagenic potential of sublethal necroptotic signaling has not yet been directly investigated. Smac mimetics drugs, which activate apoptotic or necroptotic cell death, do not induce mutations but the mechanistic basis for this lack of mutagenic activity has not been determined. In this study, we compared the mutagenic potential of these two cell death pathways by engineering cells to activate either apoptotic or necroptotic signaling by exposing them to Smac mimetics with or without TNFα, and/or enforcing or preventing expression of apoptotic or necroptotic regulators. We discovered that sublethal concentrations of Smac mimetics in contexts that activated apoptotic signaling provoked DNA damage and mutations in surviving cells. Mutagenesis was dependent on executioner caspase activation of the nuclease CAD. In contrast, RIPK3- and MLKL-dependent necroptotic signaling following Smac mimetic treatment was not mutagenic. Likewise, DNA damage was not provoked in cells expressing a lethal constitutively active MLKL mutant. These data reveal that cells surviving sublethal necroptotic signaling do not sustain genomic damage and provide hope for a reduced risk of therapy-related malignancies in patients treated with necroptosis-inducing drugs.

## Introduction

Conventional chemotherapy drugs induce DNA damage and genotoxic stress by generating DNA crosslinks, adducts or strand breaks to hopefully initiate a DNA damage response leading to p53-mediated intrinsic apoptosis of malignant cells^1^. Some clinically employed and experimental anticancer agents such as BH3 mimetics or death ligands directly trigger proapoptotic functions by deactivating the prosurvival properties of Bcl-2-like proteins or activating death receptor-mediated extrinsic apoptotic signaling, respectively^2,3^. Unfortunately, DNA damaging therapies can introduce mutations in cells that fail to die and mis-repair their DNA, with drugs exhibiting varied mutagenic potential depending on the type of lesions generated^4^. Such therapies can therefore spur the formation of second malignancies, known as “therapy-related” cancers, in cured ex-patients particularly in individuals with germline defects that compromise the recognition or repair of DNA damage^5,6^. Furthermore, direct activation of apoptotic pathways via sublethal caspase activity can also be mutagenic^7–9^. Cleavage of ICAD by executioner caspases-3/7 enables the release and activation of the nuclease CAD^10^, which generates double stranded DNA breaks that are recognized by the cell’s DNA damage response system^11,12^. The mis-repair of CAD-mediated DNA strand breaks in cells that avoid a cell death fate following caspase activation highlights the mutagenic and potentially oncogenic consequence of sublethal apoptotic signaling^13^.

Inhibitor of apoptosis proteins (IAPs) are a class of prosurvival proteins that are often overexpressed in cancers, rendering many tumors resistant to chemotherapies^14^. Compounds antagonizing the prosurvival properties of IAPs have been designed to imitate the N-terminal region of the cellular Smac/Diablo protein and as such these “Smac mimetics” display high affinity for cellular IAP 1 and 2 (cIAP1/2) and/or X-linked IAP (XIAP), but may also target other IAPs with low affinity^15^. Safe dosing regimens for a number of these compounds including LCL161, GDC0152, AT406 and Birinapant as single agents or in combination with other therapeutics have been determined, and subsequent clinical trials are ongoing^16–20^. Smac mimetics can activate two main forms of cell death: apoptosis or necroptosis. These drugs promote the auto-degradation of cIAP1/2, which normally act as E3 ubiquitin ligases to polyubiquitinate receptor-interacting protein kinase-1 (RIPK1) and establish NFκB activation of prosurvival pathways upon TNF receptor 1 (TNFR-1) ligation^21^. Non-ubiquitinated RIPK1 can then interact with FADD to activate pro-caspase-8, which stimulates executioner caspase activity and thus apoptosis. Noncanonical NFκB-mediated TNFα production can also occur in some cell types following cIAP1/2 degradation to provide autocrine TNFR-1 stimulation and enhanced cell death propagation^22^. XIAP is a potent caspase-3, -7 and -9 inhibitor and mimetics can also relieve XIAP-mediated suppression of these proteases to allow apoptosis execution^23,24^. Conversely, necroptosis can be initiated in cells deficient in caspase-8 function. In this context, RIPK1 associates with its homolog receptor-interacting protein kinase-3 (RIPK3) via their RHIM domains to form the necrosome complex, activating RIPK3 which phosphorylates the pseudokinase MLKL (mixed lineage kinase domain-like protein)^25^. Active MLKL oligomerizes and translocates to the cell surface where plasma membrane pores are formed leading to the execution of necroptotic cell lysis^26,27^.

While necroptosis induction in vitro has been well characterized to require a concoction of TNFα, Smac mimetic (to deplete cIAP1/2) and a caspase-8 inhibitor, the activation of other death receptors, Toll-like receptors, or RHIM-domain-containing proteins have also been described to mediate this mode of cell death^28^. The utility of necroptosis in vivo has been proposed as an alternative mode of cell death to enable the destruction of apoptosis-resistant pathogen infected cells, particularly to counteract the prosurvival action of viruses that suppress caspase-8-mediated apoptosis^29^. As such, necroptosis is referred to as a pro-inflammatory form of caspase-independent cell death, evoking immune activation to aid in the clearance of compromised cells^30^.

Given the absence of apoptotic caspases in necroptotic cell death signaling, we reasoned that sublethal levels of necroptosis achieved would not mutate surviving cells, and may avoid the mutagenesis associated with direct DNA damage or sublethal apoptotic signaling. We manipulated cells to respond to Smac mimetics by either activating apoptotic or necroptotic pathways then assessed the induction of DNA damage and acquisition of mutations in clonogenically competent surviving cells. Mutagenesis at the hypoxanthine-guanine phosphoribosyltransferase (HPRT) locus was quantified by growing cells that received apoptotic or necroptotic stimuli in the presence of cytotoxic 6-thioguanine (6-TG), which suppresses growth of cells expressing functional HPRT but allows the proliferation and colony formation of HPRT defective cells due to drug-induced loss-of-function mutations^31^. These cells are hence 6-TG resistant. Unlike Smac mimetic-induced apoptotic signaling, activation of RIPK3- and MLKL-dependent necroptotic signaling failed to provoke DNA damage or mutations in surviving cells.

## Materials and methods

### Cell lines and reagents

LN18 cells were purchased from ATCC, SV-40 transformed mouse embryonic fibroblasts (MEF) were kindly gifted by Anissa Jabbour and Paul Ekert, and U937 cells kindly gifted by James Murphy. LN18 and MEF cells were cultured in Dulbecco’s modified Eagle medium with high glucose (Invitrogen; CA, USA), and U937 cells were cultured in RPMI-1640 containing HEPES buffer (Invitrogen). All media was supplemented with 10% FBS (Scientifix Life; VIC, Australia) and cells maintained at 37 °C in air supplemented with 5% CO_2_. LN18 cells stably expressing FLAG-MBP (clones 2.1 and 2.2) or FLAG-CrmA (clones 1.4 and 1.5), or LN18 and U937 Cas9 control and CRISPR generated knockout cells deficient in MLKL (clones 4 and 31), RIPK3 (clones 9 and 13) or caspases-3/7 (C3/7 clones 2.5 and 1.7, or 2 and 8) have been previously described^32^.

The following drugs were used: AT406/Debio1143 (Selleck Chemicals; TX, USA), Birinapant (ApexBio; TX, USA), GDC0152 (Selleck), LCL161 (Selleck), soluble TRAIL (Peprotech; NJ, USA), doxycycline (Sigma; MO, USA), doxorubicin (Selleck) and cisplatin (Sigma). Other reagents include human and murine TNFα (Peprotech), Q-VD-OPh (R&D Systems; MN, USA), necrostatin-1 (Sigma) and GW806742X (Sigma). The following antibodies were used: rabbit anti-phospho-MLKL Ser358 (Cell Signaling Technology; MA, USA; #91689), rat anti-MLKL clone 3H1 (gift from James Murphy), rabbit anti-RIPK3 (Cell Signaling Technology; #13526), mouse anti-PARP (Cell Signaling Technology; #9532), rabbit anti-phospho-H2AX Ser139 clone 20E3 (Cell Signaling Technology; #9718), mouse anti-FLAG (M2) (Sigma; #3165), mouse anti-GAPDH (Merck Millipore, MA, USA; MAB374), donkey anti-rabbit-HRP (GE Healthcare Life Sciences; NJ, USA; NA934), goat anti-rat-HRP (GE Healthcare Life Sciences; NA935), goat anti-rabbit-FITC (Merck Millipore; AQ132F), and rabbit anti-mouse-HRP (Sigma; A9044).

### Plasmids

The pEF-FLAG-MBP^11^, pEF-FLAG-ICAD^D117, D224E^ (uncleavable ICAD mutant)^11^ and pEF-FLAG-CrmA^32^ plasmids have been described previously. The pCW57-GFP-2A-MCS was a gift from Adam Karpf (Addgene plasmid #71783; http://n2t.net/addgene:71783; RRID:Addgene_71783)^33^. This plasmid was cut with BamHI and EcoRI, dephosphorylated and used to clone the following products in order to generate doxycycline inducible expression plasmids. Human RIPK3 coding sequence was amplified from U937 cDNA using primers: 5′-GCCAATTGACCATGTCGTGCGTCAAGTTATGGCC-3′ and 5′-GCAGATCTTTATTTCCCGCTATGATTATAC-3′, and cut with MfeI and BglII. The N-terminal region (corresponding to the first 201 amino acids) of human MLKL was amplified using primers: 5′-GCGAATTCACCATGGAAAATTTGAAGCATATTATC-3′ and 5′-TTAAGATCTCTAAAGCTGCTCCTTCTTGATCTCC-3′, and cut with EcoRI and BglII.

### Stable transfection

Stable LN18 transfectants were generated using the FuGENE HD transfection reagent (Roche; Basel, Switzerland) as per manufacturer’s instructions. After 2 days in fresh media, cells were selected in 3 µg/ml puromycin (Sigma) and colonies picked for characterization after 14–18 days. Stable U937 transfectants were generated by Nucleofection (Lonza; NJ, USA). One million U937 cells, 800 ng of plasmid DNA and Nucleofector SF solution were combined then nucleofection performed using the DN-100 program on a Nucleofector device (Lonza). After 2 days growth in RPMI media containing 20% FBS, cells were selected in 1 µg/ml puromycin by seeding at 1000 cells/well in round bottom 96-well plates and grown for 14–18 days before expanding for characterization.

### Cell death and cell survival

Cell death was determined by flow cytometry. Cells were incubated with drugs or media for specified times then harvested and resuspended in binding buffer (10 mM HEPES, 140 mM NaCl, 2.5 mM CaCl2; pH 7.4) containing 1:200 of annexin-V-FITC (Abcam, Cambridge, UK) or annexin-V-V450 (BD Biosciences; CA, USA) depending on the experiment. Cells were incubated for 10 min at room temperature then an equal amount of binding buffer containing 2 µg/ml propidium iodide (Sigma) was added. Flow cytometric analysis was conducted using a FACS Canto II (BD Biosciences).

Clonogenic assays were performed to assess the ability of surviving cells to proliferate^34^. Following treatment, cells were washed once in PBS, counted and seeded at 100, 300, or 1000 cells per 6-well. Cells were stained with methylene blue (Sigma; 1.25 g/L in 50% methanol) after 7 days for MEFs or 10–12 days for LN18 cells, and the number of colonies counted.

ATP activity in cells was measured using the CellTiter-Glo 2.0 assay kit (Promega; WI, USA) to provide a readout of cell viability. Two thousand MEF or 10,000 U937 cells were seeded in white 96-well plates containing drugs or media and incubated for 24 h. CellTiter-Glo reagent was added to wells at a ratio of 1:3 and plates incubated for 10 min at room temperature before luminescence detection. Luminescence was measured using a Spectromax M5e (Molecular Devices; CA, USA).

### Caspase activity assay

DEVDase activity in cells was determined using the Caspase-Glo 3/7 assay kit (Promega). Two thousand LN18 or MEF cells, or 10,000 U937 cells were seeded in media alone or media containing drug into white 96-well plates and incubated for specified times. Caspase-Glo 3/7 reagent was added as 1:2 to each well and plates incubated at room temperature for 30 min prior to luminescence detection using a Spectromax M5e (Molecular Devices).

### γH2AX detection by flow cytometry

Following drug treatment, cells were harvested and fixed in cold 70% ethanol at −20 °C overnight. The next day, ethanol was removed by centrifugation and cells rehydrated in TXT buffer (4% FBS and 0.1% Triton-X100 in TBS). Cells were stained with rabbit anti-phospho-H2AX Ser139 in TXT buffer (1:200) for 2 h shaking at room temperature, washed with TBS then stained with anti-rabbit-FITC antibody in TXT buffer (1:200) for 1 h shaking at room temperature. After washing with TBS, cells were finally resuspended in TBS containing 1 µg/ml propidium iodide and kept on ice until analysis of the FITC-positive signal of cells in G0/1 phase by a FACS Canto II (BD Biosciences).

### HPRT assay

HPRT mutagenesis was determined by scoring the amount of colony growth in 6-thioguanine (6-TG; Sigma)^35^. Ten (MEF) or 20,000 (LN18) cells were seeded in 24-wells a day before treatment. Following treatment, cells were washed with PBS and cultured for 7 days to ensure any residual HPRT enzymatic function was lost^36^, recounted with trypan blue to exclude dead cells then 3 × 10^5^ cells seeded in media containing 6-TG (1 or 30 µM, MEF or LN18 cells respectively) in 15 cm culture dishes. Colonies were stained with methylene blue (1.25 g/L in 50% methanol) after 7 (MEF) or 18 (LN18) days.

### Immunoblotting

Cells were lysed using RIPA lysis buffer (150 mM sodium chloride, 1.0% Triton X-100, 0.5% sodium deoxycholate, 0.1% SDS, 50 mM Tris, pH 8.0, supplemented with protease inhibitor cocktail; Sigma) by pipetting 50 times and rapid vortexing. The lysates were cleared by centrifuging for 15 min at 16,100*g* at 4 °C. Total protein was determined using the bicinchoninic acid (BCA) method (Micro BCA Protein assay kit, Thermo Fisher Scientific; MA, USA). Fifty micrograms of lysates were loaded on tris-glycine gels and the proteins were separated by sodium dodecyl sulfate-polyacrylamide gel electrophoresis then transferred onto Hybond PVDF 0.22 µm membrane (Millenium Science; Victoria, Australia). Membranes were blocked with 1% blocking reagent (Roche) in phosphate-buffered saline (PBS), and probed with primary antibodies then horseradish peroxidase (HRP)-conjugated secondary antibodies diluted in 1% blocking reagent (Roche) in PBS with 0.1% Tween-20 (Sigma). SuperSignal West Dura extended duration substrate (Thermo Fisher Scientific) was used for detection.

### Statistics

GraphPad Prism 8.0 was used to perform one-way or two-way ANOVA analyses (specified in the figure legends) with Sidak post-tests to assess the significance of differences in responses of untreated and treated cells. All experiments were independently repeated three times and data presented as the mean value with error bars representing SEM from biological replicates.

## Results

### Caspase-deficient cell death signaling does not provoke mutations

**Fig. 1:**
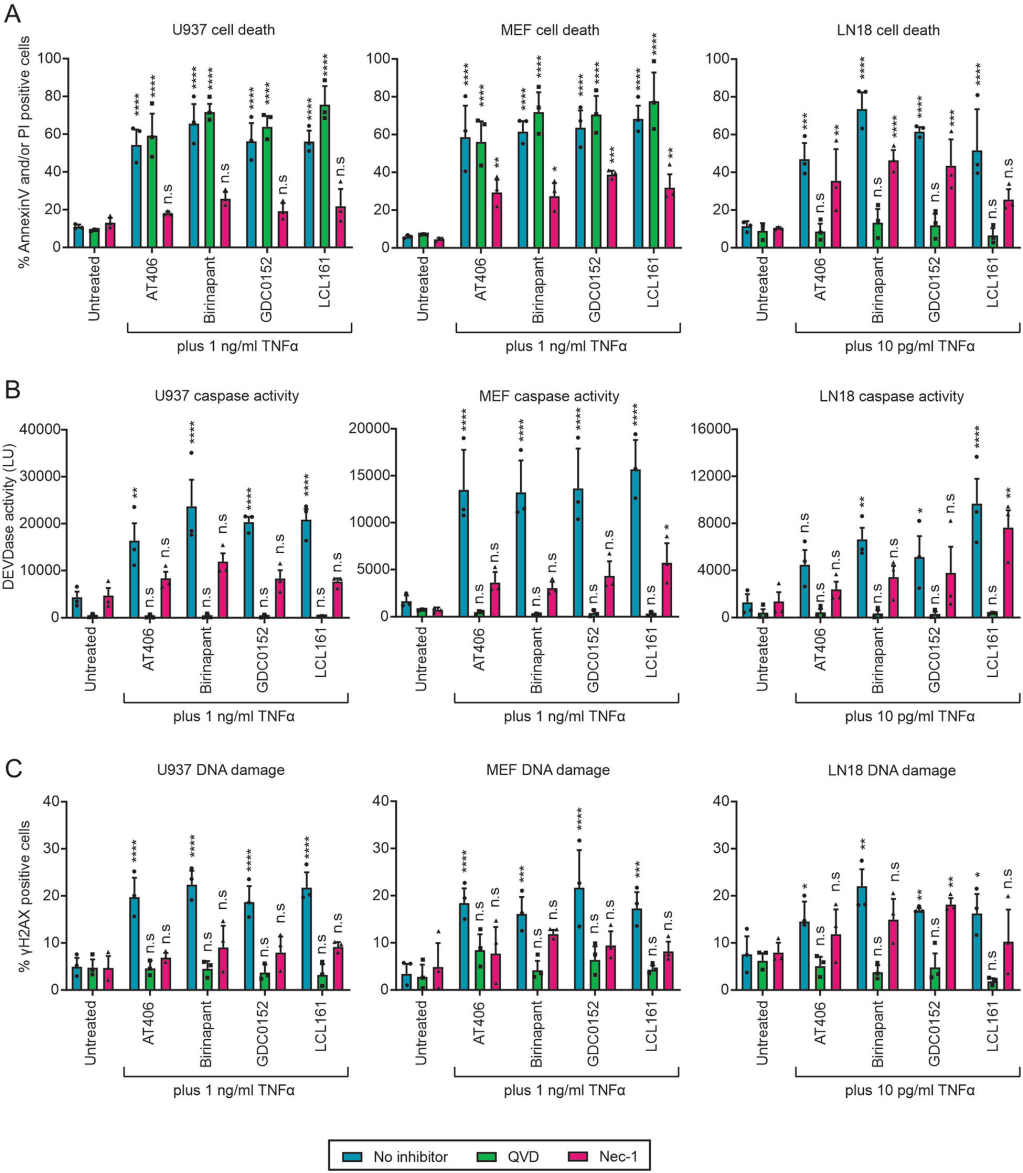
Caspase-independent death is not associated with DNA damage. U937, MEF, or LN18 cells were treated with the indicated amount of TNFα plus 10 µM Smac mimetic (AT406, Birinapant, GDC0152 or LCL161) following 1 h pretreatment or not with 10 µM Q-VD-OPh (QVD) or necrostatin-1 (Nec-1). **a** Cell death was determined after 24 h, while **b** DEVDase activity and **c** the proportion of cells experiencing DNA damage was determined after 6 h. Two-way ANOVA with Sidak post-tests were used to estimate the probability that random chance accounted for the differences observed between untreated and treated cells (*p < 0.05; **p < 0.01; ***p < 0.001; ****p < 0.0001; ^ns^*p* > 0.05; data represent mean ± SEM from three independent biological replicates).

We assayed three cell lines of different lineages to assess the ability of TNFα co-treatment with Smac mimetics to provoke DNA damage or mutate cells at the HPRT locus. Treatment of U937, MEF, or LN18 cells with TNFα combined with various Smac mimetics enabled annexin-V binding to phosphatidylserine and/or membrane permeabilization (Fig. 1a), enhanced DEVDase levels (Fig. 1b), and induced DNA damage as indicated by H2AX phosphorylation (γH2AX) (Fig. 1c). The addition of the pan caspase inhibitor Q-VD-OPh (QVD) suppressed DEVDase activity and the ensuing DNA damage following treatment in all cell types, implying DNA damage coincided with caspase activity, but only spared LN18 cells from death indicating caspase-independent death was achieved in U937 and MEF cells. Furthermore, TNFα/Smac mimetic signaling occurred via RIPK1 in U937 and MEF cells as necrostatin-1 (Nec-1) reduced cell death and DEVDase activity in these cells. RIPK1 inhibition by Nec-1 also inhibited the DNA damage provoked by co-treatment with Smac mimetics plus TNFα.

**Fig. 2:**
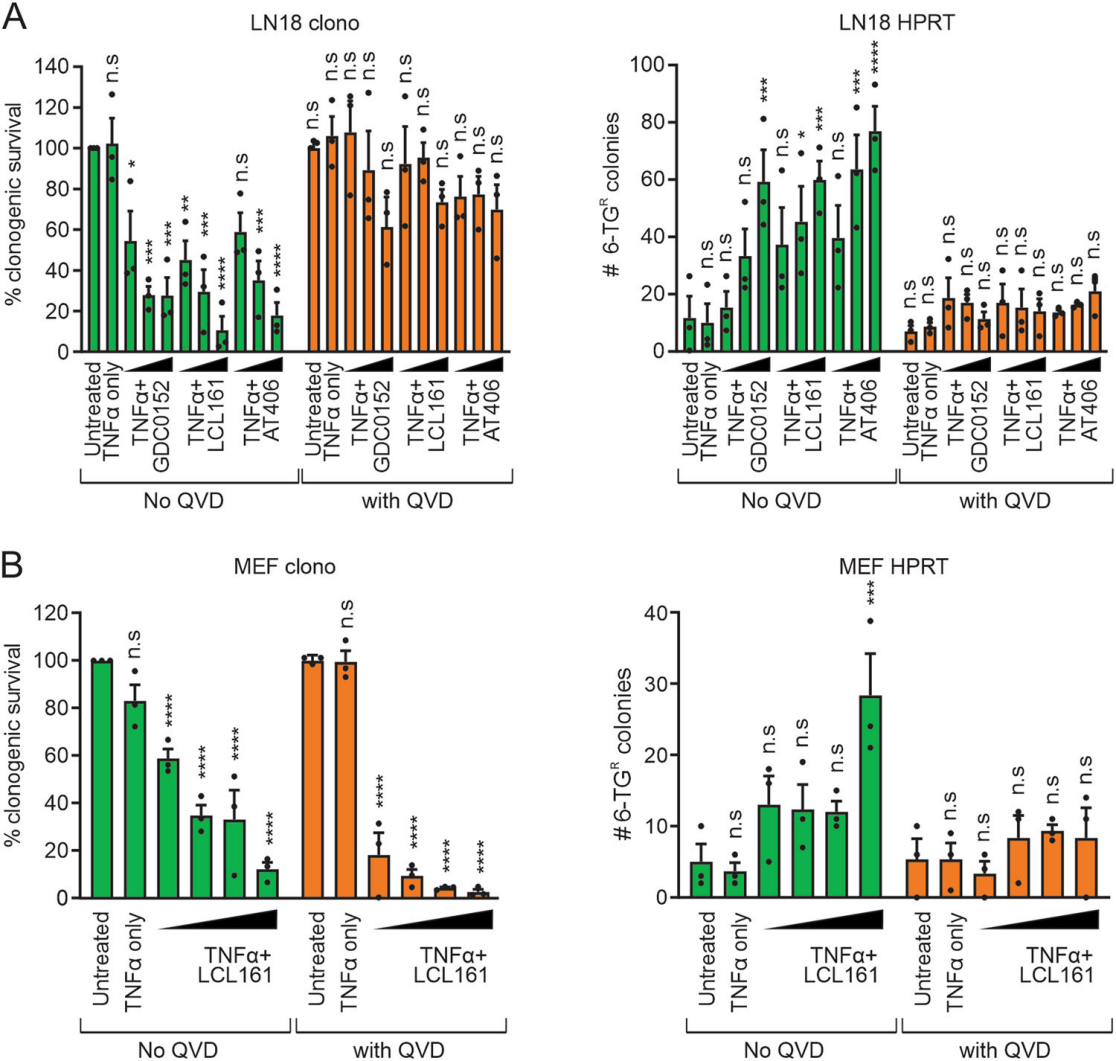
Co-treatment of TNFα and Smac mimetics provoke HPRT mutations but only under caspase proficient conditions. **a** LN18 cells were incubated or not with Smac mimetics (0.3, 1, or 3 µM) in the presence of 10 pg/ml TNFα, while **b** MEF cells were exposed to 1 ng/ml TNFα plus LCL161 (0.3, 1, 3, or 10 µM). Some cells were pretreated with 10 µM QVD. Following 24 h, the clonogenic potential was determined (left panels) and the surviving cells grown in 6-TG to select for the emergence of HPRT mutant colonies (right panels). Two-way ANOVA with Sidak post-tests were used to estimate the probability that random chance accounted for the differences observed between untreated and treated cells (*p < 0.05; **p < 0.01; ***p < 0.001; ****p < 0.0001; ^ns^*p* > 0.05; data represent mean ± SEM from three independent biological replicates).

Mutation assays were then performed to determine if the DNA damage we observed resulted in mutations at the HPRT locus, and whether the lack of γH2AX induction under conditions of caspase-independent cell death reflected a lack of mutagenesis. Incubation of LN18 or MEF cells with DNA damaging chemotherapy agents impaired clonogenicity and enabled colony formation in the presence of 6-TG (Supplementary Fig. 1), implying HPRT mutations and validating this assay. TNFα alone did not affect clonogenic survival or increase the frequency of 6-TG resistant cells but more colonies emerged when TNFα was combined with Smac mimetics implying that, unlike non-TNFα-stimulated Smac mimetic exposure (Supplementary Fig. 1a), the combination treatment provoked HPRT mutations (Fig. 2). As expected, pretreatment with QVD protected LN18 cells from TNFα/Smac mimetic-induced clonogenic death and prevented the emergence of 6-TG resistant colonies (Fig. 2a). This effect was further observed in cells deficient in caspases-3 and -7 (C3/7 DKO) as these cells only displayed background levels of γH2AX induction or 6-TG resistant colonies following TNFα/LCL161 treatment (Supplementary Fig. 2). Interestingly, incubation with TNFα/LCL161 impaired clonogenicity of C3/7 DKO lines but to a lesser degree than Cas9 control (Supplementary Fig. 2b). The incomplete protection afforded by loss of the executioner caspases most likely reflects mitochondrial impairments upstream of executioner caspases that impacted on clonogenicity, rather than necroptosis as these cells lack RIPK3 expression^32^. TNFα combined with LCL161 also impeded the clonogenic potential of MEF cells in a dose dependent manner, and this effect was maintained even when caspases were inhibited by QVD (Fig. 2b). More 6-TG resistant colonies were formed from MEF cultures that survived TNFα/LCL161 exposure in the absence QVD pretreatment.

Prior research has described the ability of the caspase activated nuclease CAD to damage DNA and provoke mutagenesis upon apoptotic stimuli^7,8,11,37,38^. We stably expressed a mutant version of ICAD (mICAD), which bears glutamic acid residues at the P1 position of the caspase cleavage sites to render it uncleavable by active caspases^10^, in LN18 and U937 cells to confirm the ability of TNFα/LCL161 co-treatment to damage DNA via active CAD (Supplementary Fig. 3). Similar levels of cell death and caspase activation were achieved between all stable lines, while an increase in the number of cells bearing γH2AX after TNFα/LCL161 treatment was only detected in MBP stable transfectants and not in cells expressing mICAD.

These data reveal that cells surviving TNFα and Smac mimetic co-treatment acquire DNA damage and HPRT mutations via CAD-mediated DNA damage following activation by sublethal levels of executioner caspases, and that mutagenesis does not occur under caspase-deficient conditions.

### RIPK3 and MLKL are required for non-mutagenic necroptotic signaling

Given that Smac mimetics have been documented to induce RIPK3/MLKL-mediated necroptotic cell death in cells that lack caspase-8 activity and express RIPK3^39,40^, we confirmed that necroptotic signaling was activated in U937 and MEF cells upon TNFα/LCL161/QVD treatment, and found that this did not promote mutagenesis. U937 cells deficient in either RIPK3 or MLKL^32^ were insensitive to TNFα/LCL161/QVD-induced death (Supplementary Fig. 4a), confirming classical RIPK3-/MLKL-dependent necroptotic signaling was triggered by TNFR-1 ligation while IAPs and caspase-8 were inhibited. GW806742X was used to confirm the requirement for MLKL in TNFα/LCL161/QVD-induced death in MEF cells, as this compound suppresses MLKL activity by targeting the pseudokinase domain of murine MLKL^41^. GW806742X maintained the viability of MEF cells treated with TNFα/LCL161/QVD and (to a lesser extent) TNFα/LCL161 (Supplementary Fig. 4b). The slight protection of cells treated with TNFα/LCL161 could be due to chance or possibly due to minor affinity of the inhibitor for RIPK1^41^.

Cells that were sensitive to necroptotic conditions (Cas9 or C3/7 deficient cells) did not exhibit H2AX phosphorylation, while an increase in cells experiencing DNA damage was observed only in cells that were proficient in caspase-3/7 activity and treated with TNFα/LCL161, regardless of RIPK3 or MLKL expression (Supplementary Fig. 4c). Phosphorylated H2AX was detected in TNFα/LCL161-treated lysates that also contained cleaved PARP, indicating caspase activation, but not in lysates of TNFα/LCL161/QVD-treated cells that contained phosphorylated (and therefore active) MLKL and lacked PARP cleavage (Supplementary Fig. 4d). These experiments confirm that TNFα/LCL161/QVD treatment activates MLKL-mediated necroptosis in U937 and MEF cells, and that this pathway does not damage DNA.

**Fig. 3:**
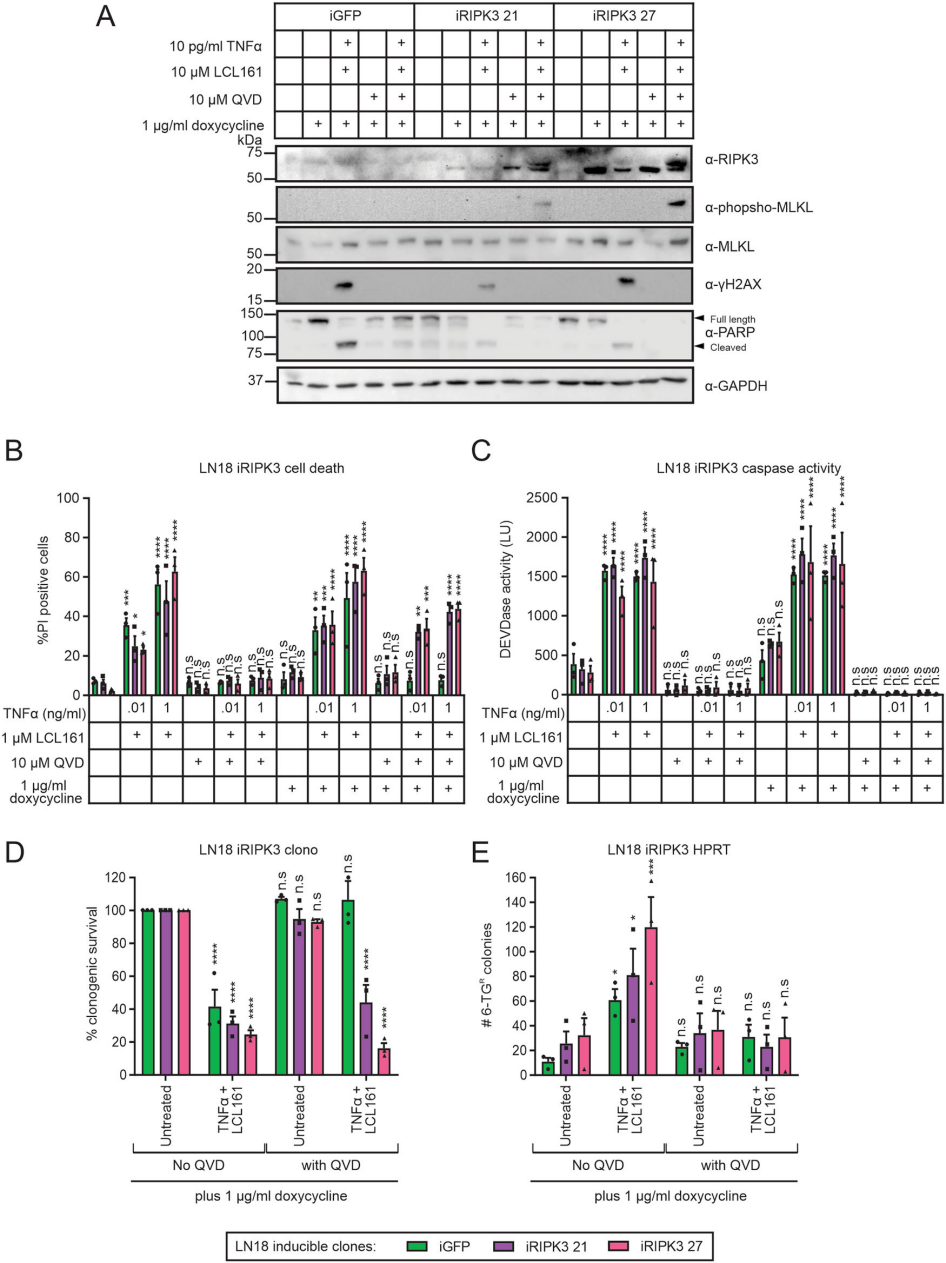
Inducible expression of RIPK3 sensitizes LN18 cells to necroptosis which is not mutagenic. LN18 cells transfected with a GFP or RIPK3 inducible expression plasmid were grown in doxycycline (to induce expression) for 24 h prior to treatment, then treated with TNFα, LCL161, and/or QVD (1 h pretreatment). **a** Immunoblotting was performed to confirm RIPK3 expression and determine phosphorylated and total levels of MLKL, phosphorylated H2AX, full length and cleaved PARP, or GAPDH. **b** PI uptake was used to determine the proportion of dead cells and **c** DEVDase activity also measured. RIPK3 expressing cells treated with 10 pg/ml TNFα plus 1 µM LCL161, with 10 µM QVD pretreatment or not, were assessed for **d** changes in clonogenic potential and **e** the ability of surviving cells to grow and form colonies in 6-TG. Two-way ANOVA with Sidak post-tests were used to estimate the probability that random chance accounted for the differences observed between untreated and treated cells (*p < 0.05; **p < 0.01; ***p < 0.001; ****p < 0.0001; ^ns^*p* > 0.05; data represent mean ± SEM from three independent biological replicates).

LN18 cells did not succumb to TNFα/Smac mimetic-mediated cell death in the presence of QVD most likely because these cells do not express RIPK3 thereby prohibiting MLKL activation via the necrosome^32,39^. To sensitize LN18 cells to necroptosis, we engineered these cells to contain a doxycycline inducible RIPK3 expression plasmid. Cells capable of inducible GFP expression were used as a control. Non-lethal RIPK3 expression in LN18 cells was achieved when cells were grown in 1 µg/ml doxycycline (Fig. 3a). TNFα/LCL161 treatment of LN18 cells expressing RIPK3, or not, resulted in cleavage of PARP, cell death and enhanced DEVDase levels (Fig. 3a–c), indicating caspase-dependent death. As expected, phosphorylated H2AX was also detected in these cells. GFP expressing cells treated with TNFα/LCL161 in the presence of QVD lacked PARP cleavage, remained viable (Fig. 3b) and did not display DEVDase activity (Fig. 3c) or DNA damage (Fig. 3a). In contrast, RIPK3 expressing cells treated under the same conditions displayed phosphorylation of RIPK3 (as indicated by the upper band in doublet^42^) and MLKL but not H2AX (Fig. 3a). RIPK3 expression also enabled the killing of LN18 cells by TNFα/LCL161/QVD treatment (Fig. 3b) and this occurred in the absence of DEVDase activity (Fig. 3c). These data confirm the activation of necroptosis in RIPK3-expressing LN18 cells treated with TNFα/LCL161/QVD. Unlike GFP, inducible expression of RIPK3 impaired clonogenicity following TNFα/LCL161/QVD treatment (Fig. 3d) and this was not associated with an increase in the number of 6-TG resistant colonies (Fig. 3e). Exposure to TNFα/LCL161 in the absence of QVD provoked more 6-TG resistant colonies in GFP or RIPK3 expressing cells. This demonstrates that expression of RIPK3 was required to enable necroptotic signaling upon TNFα/LCL161 treatment in LN18 cells under caspase deficient conditions, and revealed that this signaling does not provoke DNA damage or mutations.

**Fig. 4:**
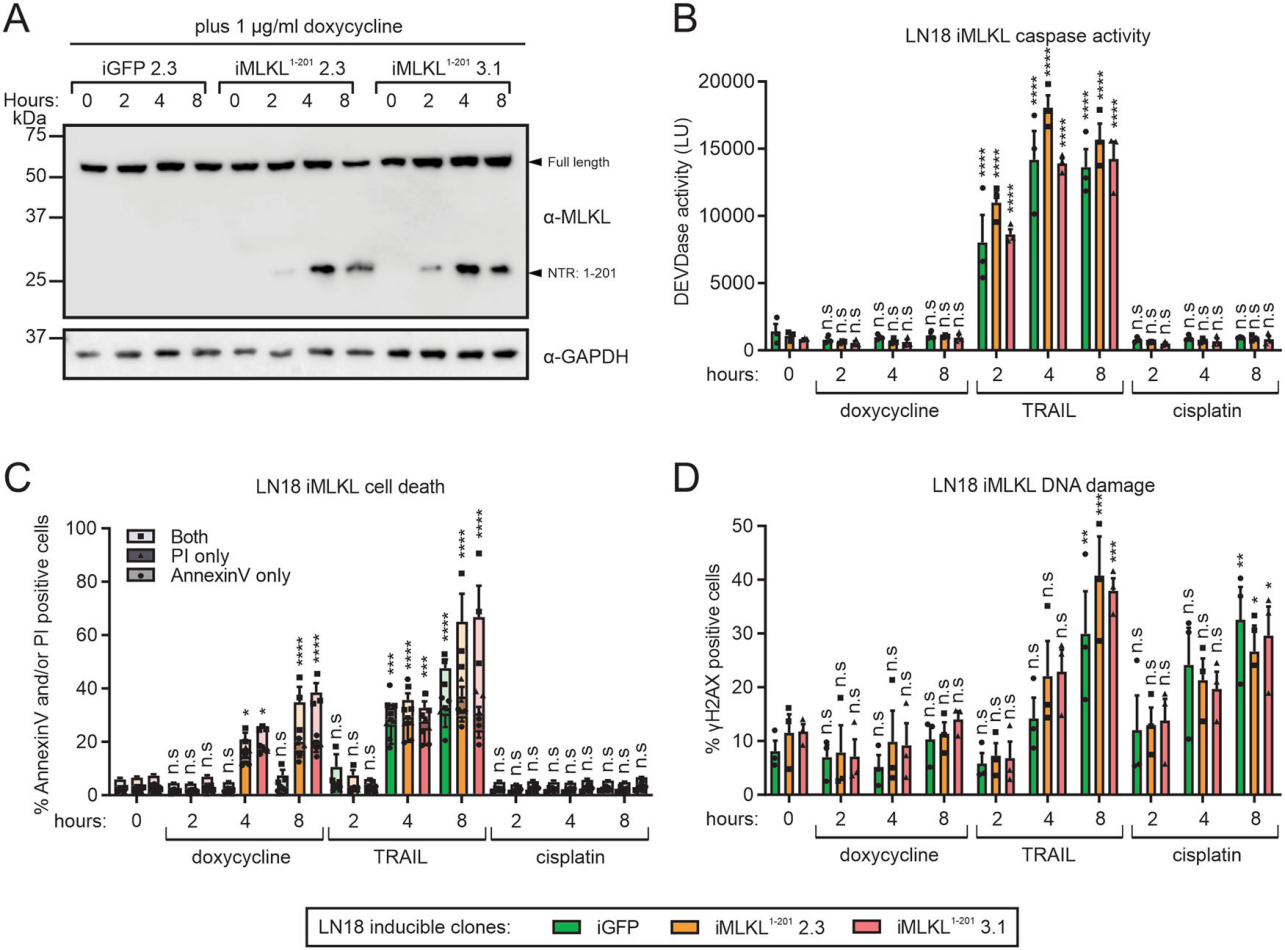
Inducible MLKL activation does not provoke DNA damage. LN18 cells transfected with a GFP or MLKL N-terminal region (MLKL^1–201^) inducible expression plasmid were grown in doxycycline (to induce expression) for the indicated time points. **a** Immunoblotting was used to confirm expression of MLKL^1–201^. Inducible clones were grown in 1 µg/ml doxycycline, 10 ng/ml TRAIL, or 1 µM cisplatin for indicated time points. **b** DEVDase activity, **c** cell death and **d** proportion of cells experiencing DNA damage were determined. Two-way ANOVA with Sidak post-tests were used to estimate the probability that random chance accounted for the differences observed between untreated and treated cells (*p < 0.05; **p < 0.01; ***p < 0.001; ****p < 0.0001; ^ns^*p* > 0.05; data represent mean ± SEM from three independent biological replicates).

To directly link the non-mutagenicity of necroptotic signaling to MLKL-mediated death, we used the doxycycline inducible expression system to inducibly express a constitutively active N-terminal region (corresponding to the first 201 amino acids) of human MLKL (MLKL^1–201^) in LN18 cells (Fig. 4a). This was published to activate necroptosis when over-expressed^43^. Expression of MLKL^1–201^ provoked annexin-V binding and uptake of propidium iodide over time, which was not accompanied by DEVDase activity or DNA damage (Fig. 4b–d). In contrast, treatment of inducible clones in the absence of doxycycline (therefore no expression of active MLKL^1–201^) with TRAIL or cisplatin provoked an increase in the number of cells containing γH2AX. TRAIL enabled annexin-V binding to phosphatidyl serine prior to propidium iodide uptake and provoked rapid DEVDase activity, whereas cells remained viable after cisplatin exposure at these early time points, consistent with its indirect toxic mechanism^44,45^.

### Necroptosis is non-mutagenic in the context of CrmA expression

**Fig. 5:**
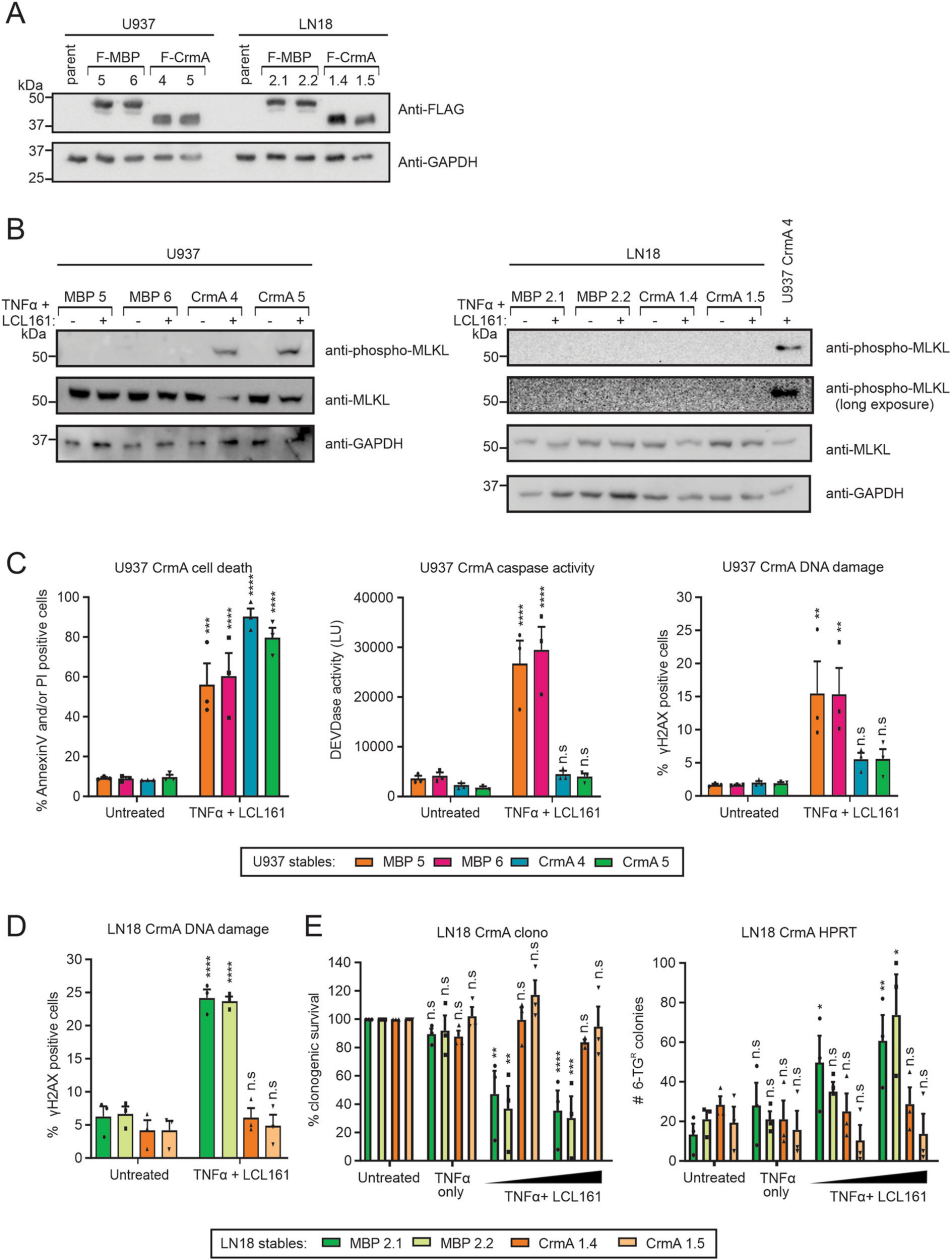
CrmA expression prevents mutagenesis following TNFα and LCL161 co-treatment. **a** Stable U937 or LN18 transfectants expressing FLAG tagged MBP or CrmA were lysed and immunoblotted. **b** Lysates were generated from cells untreated (−) or treated (+) with 1 ng/ml (U937) or 10 pg/ml (LN18) TNFα plus 10 µM LCL161 for 8 h, and immunoblotting performed to determine phosphorylated and total levels of MLKL. **c** U937 transfectants were treated with 1 ng/ml TNFα plus 10 µM LCL161 then cell death assessed after 24 h (left panel). Caspase activity (middle panel) and DNA damage (right panel) was determined after 6 h. LN18 transfectants were treated with 10 pg/ml TNFα plus LCL161 (10 µM for panel **d**, 1 or 3 µM for panel **e**) then **d** DNA damage determined after 6 h. **e** After 24 h, surviving cells were assessed for clonogenic viability (left panel) and their ability to grow and form colonies in 6-TG (right panel). Two-way ANOVA with Sidak post-tests were used to estimate the probability that random chance accounted for the differences observed between untreated and treated cells (*p < 0.05; **p < 0.01; ***p < 0.001; ****p < 0.0001; ^ns^*p* > 0.05; data represent mean ± SEM from three independent biological replicates).

The data presented so far has utilized QVD to chemically inhibit caspase-8 (as well as other caspases) in order to shift TNFR-1 signaling from apoptosis to necroptosis. To confirm that necroptotic signaling was also not mutagenic under conditions whereby a chemical caspase inhibitor was not present, we stably expressed CrmA (a poxviral Spi2 protein that can efficiently inhibit caspase-8^46^) in U937 and LN18 cells to incapacitate caspase-8 and promote TNFα/LCL161-mediated necroptosis without the requirement for QVD addition (Fig. 5a). TNFα/LCL161 treatment provoked MLKL phosphorylation in U937 cells expressing CrmA but not in MBP expressing lines (Fig. 5b). Conversely, CrmA expression in LN18 cells did not enable phosphorylation of MLKL upon TNFα/LCL161 exposure due to a lack of basal RIPK3 expression^32^. U937 CrmA stable transfectants were killed by TNFα/LCL161 but, unlike MBP stable transfectants, this treatment was not accompanied by enhanced DEVDase activity nor did it increase the proportion of γH2AX positive cells (Fig. 5c). TNFα/LCL161 treatment also failed to provoke H2AX phosphorylation in LN18 CrmA stable transfectants (Fig. 5d). These cells also maintained clonogenic viability despite TNFα/LCL161 exposure and, unlike MBP-expressing cells, only background numbers of 6-TG resistant colonies were detected following treatment (Fig. 5e). These data indicate that inhibition of caspase-8 by CrmA sensitizes U937 (but not LN18) cells to necroptosis induced by TNFα/LCL161, and CrmA expression in either cell type protects from the mutagenesis associated with TNFα/LCL161 treatment.

## Discussion

Defining the molecular components and mechanisms of various modes of cell death has been a key focus in recent years to further understand disease, such as cancers and inflammatory disorders, with the intention of developing therapeutics to effectively manipulate these cell death pathways. This is emphasized by the need to effectively eliminate cancer cells that have acquired chemo-resistance to apoptotic cell death provoked by many conventional chemotherapeutics^47^. The inaccurate repair of DNA lesions generated by genotoxic therapies can introduce mutations in non-cancerous cells to facilitate oncogenic transformation or enhance intratumoural heterogeneity in a subpopulation of apoptosis-resistant clones, thereby impacting on the formation of subsequent or relapsed cancers^48,49^. Understanding the mutagenic potential of different cell death signaling pathways that are activated by various anti-cancer therapies is therefore important as non-mutagenic pathways may reduce a patient’s risk of therapy-related cancer. As knowledge of necroptotic signaling pathways improves, we sought to define the mutagenic potential of this caspase-independent cell death pathway and determine if cells acquire mutations after experiencing sublethal activation of these pathways.

Smac mimetics are commonly used in vitro to activate necroptotic pathways when combined with TNFα and an inhibitor that targets caspase-8, so we used this approach to specifically interrogate the mutagenic potential of necroptotic signaling. We observed that sublethal exposure to Smac mimetics without concurrent TNFα treatment was non-mutagenic^34,50^. We recently discovered that those treatments triggered a lytic, non-apoptotic and non-necroptotic mode of death^32^. In this study, we engineered scenarios in which cellular responses to Smac mimetics could be switched between apoptotic and necroptotic pathways, to determine the mutagenesis associated with sublethal activation of each cell death mechanism. We found that activation of classical apoptotic pathways in cells sensitive to TNFα and Smac mimetic co-treatment provoked mutations in surviving cells that maintained clonogenic competency. Using QVD to inhibit caspase activity, cells lacking executioner caspase-3/7 expression, or stable expression of a non-cleavable mutant form of ICAD, we confirmed that the mutagenesis observed occurred via the mis-repair of DNA damage generated by the CAD nuclease upon sublethal activation of executioner caspases. This adds to prior research ascribing mutagenic and oncogenic properties to sublethal apoptotic caspase signaling^7,8,11,51,52^, and assigns for the first time mutagenic potential to Smac mimetics. TNFα and Smac mimetic co-treatment plus QVD or CrmA expression killed U937 or MEF cells without activating caspases and this was dependent on the activity or expression RIPK1, RIPK3 or MLKL, confirming classical necroptotic death. Cells undergoing necroptosis lacked γH2AX (and therefore DNA damage) and surviving cells did not acquire HPRT mutations, attributing the mutagenicity of Smac mimetics to their caspase-activating function. Although drug-treated surviving cells were cultured for a standard 7 day phenotypic lag period prior to incubation in 6-TG to ensure stable expression of a HPRT mutant phenotype^36^, it is possible that a shorter incubation time could potentially reveal some HPRT mutant clones emerging after a necroptotic stimulus, given that these clones maintained colony forming potential. LN18 cells that were engineered to express RIPK3 became sensitive to necroptotic stimuli and failed to acquire HPRT mutations under caspase-independent conditions that enabled MLKL phosphorylation, whereas mutations ensued in caspase-proficient, MLKL-independent conditions. Coupled with the inability of a constitutively active MLKL mutant to provoke DNA damage, our data demonstrate that caspase-independent necroptotic cell death avoids the DNA damage and mutations associated with signaling pathways that activate caspases. These results support the onco-protective effects of caspase-independent modes of cell death^53^.

The clinical use of Smac mimetics is currently being evaluated and their ability to mutate cells in vivo, and therefore impact the risk of therapy-related cancers, remains to be determined. Our data imply that the mutagenic potential of Smac mimetics to cells of different lineages within various organs will hinge on the local concentration of TNFα and the status of caspase-8 and RIPK3 within the cells, as these factors would be expected to influence whether cells respond to IAP antagonism by activating potentially mutagenic apoptotic signaling or non-mutagenic necroptotic signaling. Pro-inflammatory cytokines are released upon activation of necroptotic proteins or through stimulation of noncanonical NFκB pathways by stabilization of NIK upon Smac mimetic treatment^54–56^. For instance, RIPK1 expression can modulate TNFα production by NFκB dependent and independent pathways^57,58^, and the secretion of chemokines and other immunoregulatory molecules occur in a RIPK3- and MLKL-dependent manner during necroptosis^59,60^. Therefore, it may be possible for the autocrine TNFα produced by circulating or tumor-infiltrating myeloid cells^61,62^, for example, in patients treated with Smac mimetics to cooperate with the drug to trigger sublethal apoptotic signaling in noncancerous cells, which may be mutagenic and potentially could lead to oncogenic transformation.

Encouragingly, we did not observe DNA damage upon induced expression of activated MLKL suggesting future drugs that directly activate MLKL (without the ability to also activate caspases or raise TNFα levels, as Smac mimetics do) may harbor low mutagenic risk. Mechanisms that enable cells to withstand modest levels of MLKL activation have been characterized offering evidence to support our findings^42,59,63–65^. Direct MLKL activation may also avoid the potential accumulation of RIPK3-driven oxidative stress upon stimulation of the necrosome as has been reported following TNFα/Smac mimetic treatment, pathogen infection or necrosome formation^66–70^. As we did not detect evidence of DNA damage (or mutations) in necroptotic cells, we speculate that any RIPK3-dependent oxidative stress formed had no genotoxic effect.

In conclusion, this study provides hope for the safety of direct necroptosis inducing anti-cancer agents developed in the future as they may reduce the risk of therapy-related second cancers in patients whose first malignancies are cured using these therapies. Further work will be needed to determine whether Smac mimetics are non-mutagenic in vivo, or whether they can cooperate with TNFα to trigger apoptotic signaling, potentially rendering them mutagenic and possibly oncogenic in vivo.

## Supplementary information

Supplementary Figure 1

Supplementary Figure 2

Supplementary Figure 3

Supplementary Figure 4
