# Supplementary figures and images for: In vitro analysis reveals necroptotic signaling does not provoke DNA damage or HPRT mutations
Source: Cell Death Dis. 2020 Aug 13;11(8):680. doi: 10.1038/s41419-020-02879-y (PMC7442655; doi:10.1038/s41419-020-02879-y)

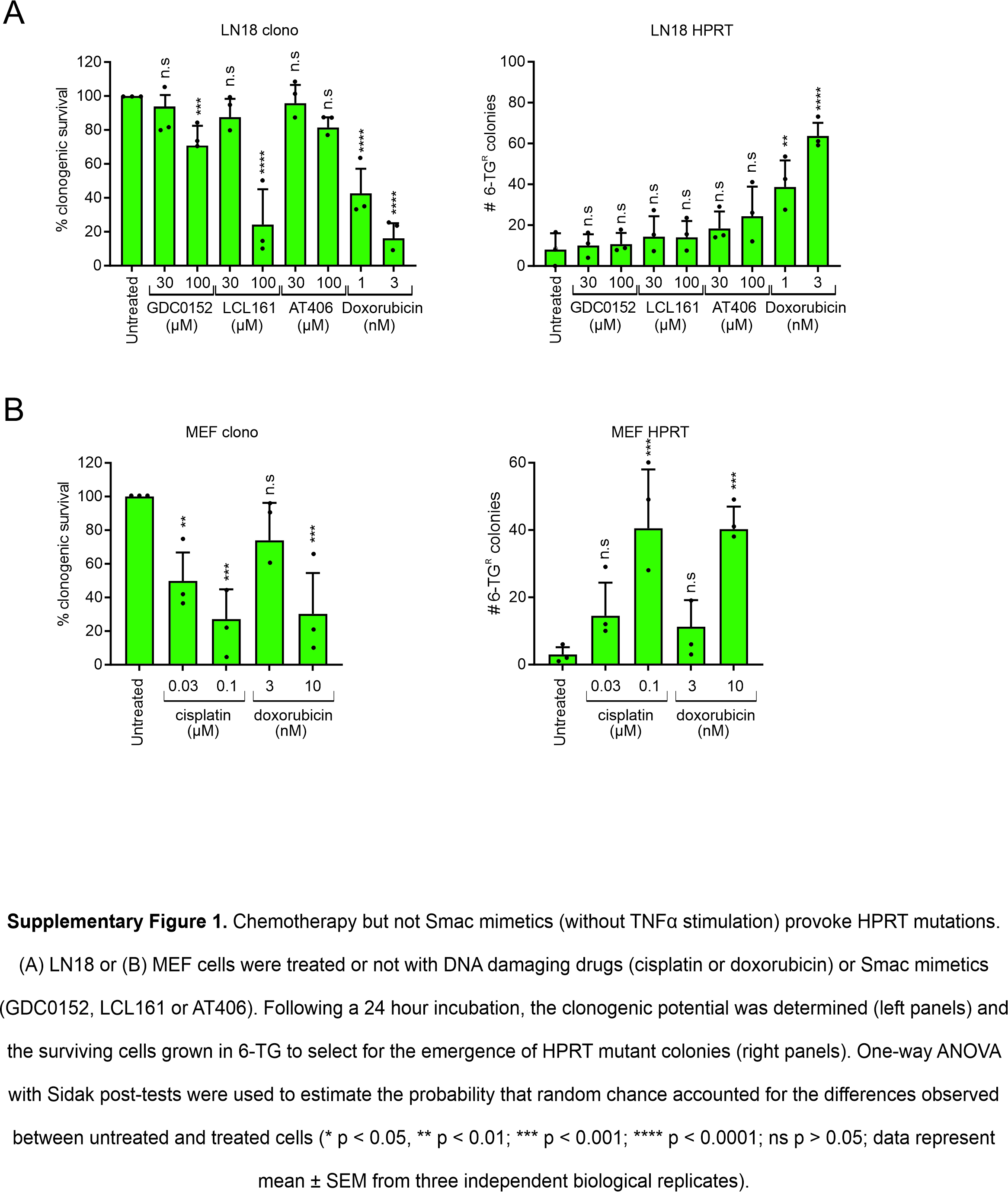

Supplement: Supplementary file 1 — Supplementary Figure 1 [file 41419_2020_2879_MOESM1_ESM.tif]

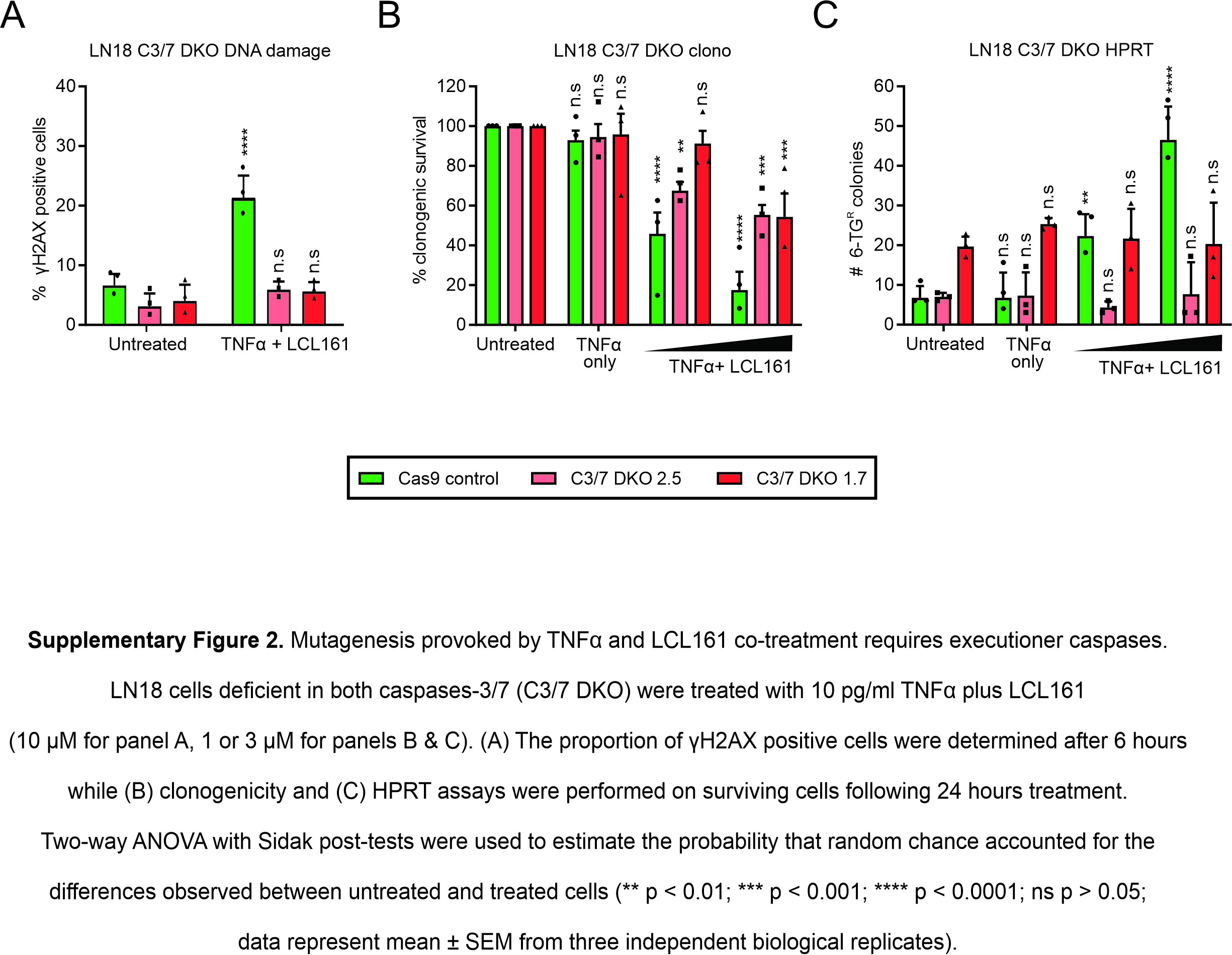

Supplement: Supplementary file 2 — Supplementary Figure 2 [file 41419_2020_2879_MOESM2_ESM.tif]

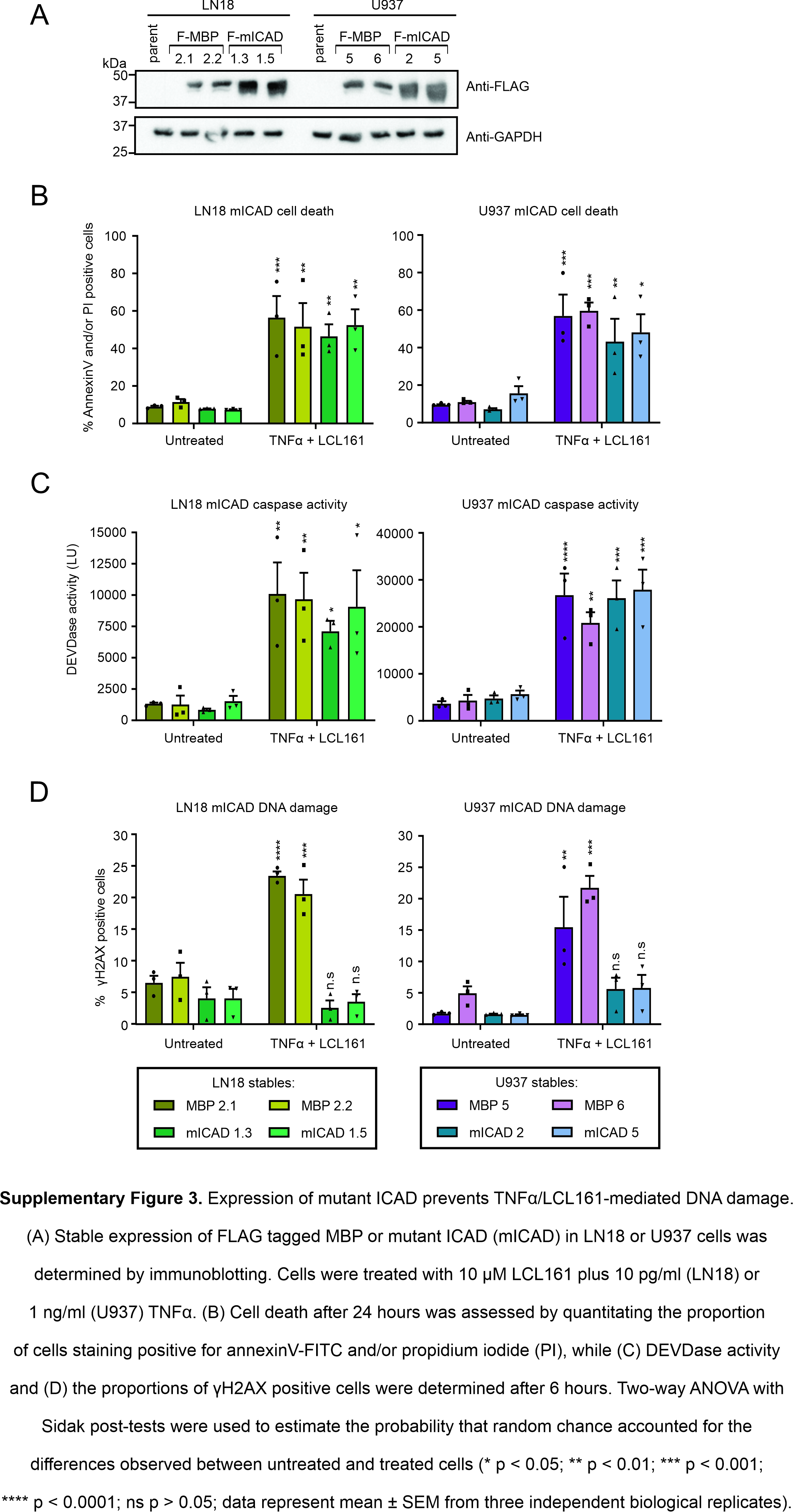

Supplement: Supplementary file 3 — Supplementary Figure 3 [file 41419_2020_2879_MOESM3_ESM.tif]

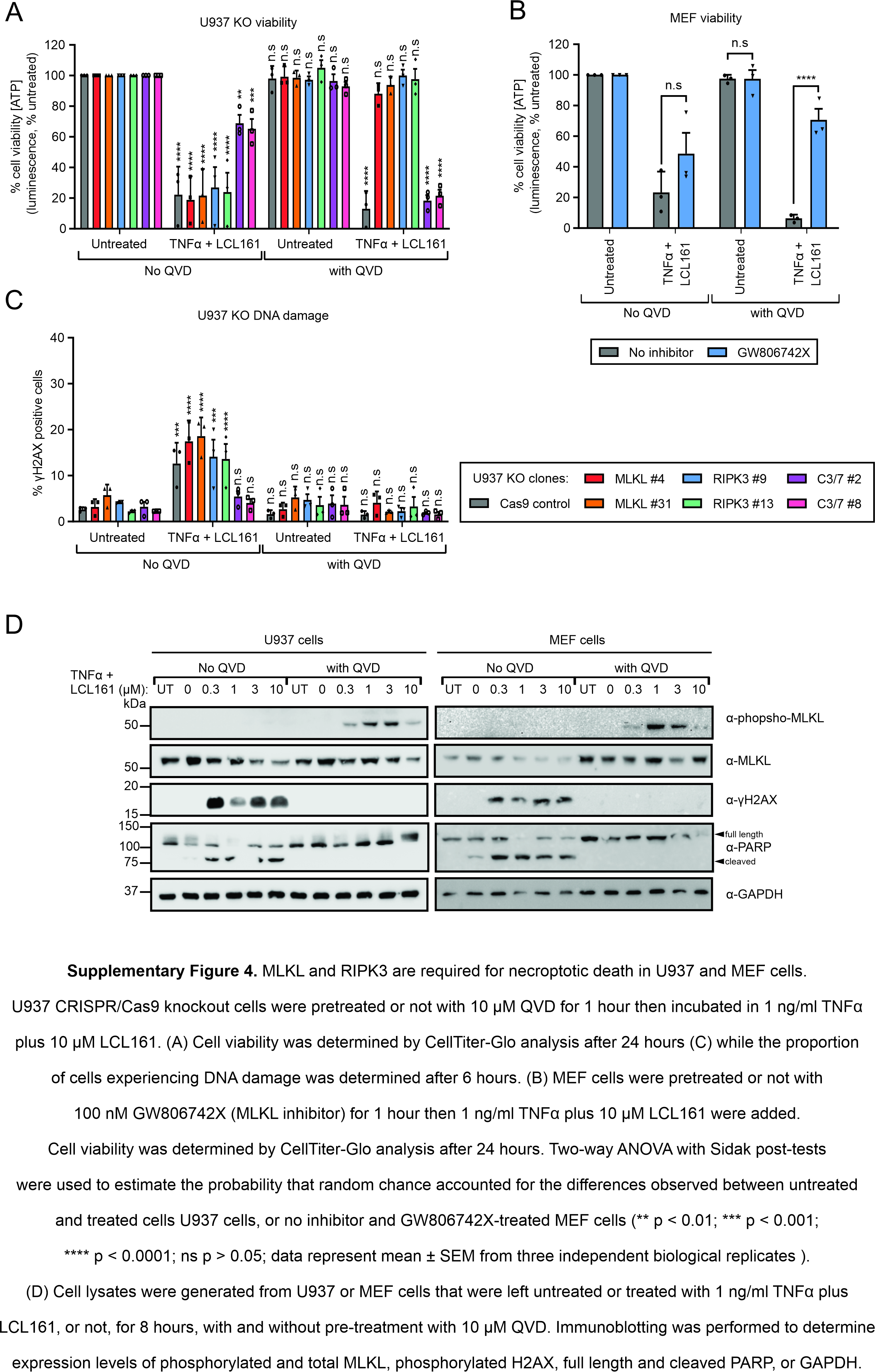

Supplement: Supplementary file 4 — Supplementary Figure 4 [file 41419_2020_2879_MOESM4_ESM.tif]
